# Supplementary material for: Global atlas of predicted functional domains in Legionella pneumophila Dot/Icm translocated effectors
Source: Mol Syst Biol. 2024 Nov 19;21(1):3. doi: 10.1038/s44320-024-00076-z (PMC11696984; doi:10.1038/s44320-024-00076-z)
Supplement: Supplementary file 1 — Appendix [file 44320_2024_76_MOESM1_ESM.pdf]

Appendix for Global atlas of predicted functional domains in *Legionella pneumophila* Dot/Icm translocated effectors

Deepak T. Patel[1], Peter J. Stogios[2], Lukasz Jaroszewski[3], Malene L. Urbanus[4], Mayya Sedova[3], Cameron Semper[1], Cathy Le[1], Abraham Takkouche[3], Keita Ichii[3], Julie Innabi[3], Dhruvin H. Patel[1], Alexander Ensminger[4,5*], Adam Godzik[3*], Alexei Savchenko[1,2*]

Affiliations:

[1] Department of Microbiology, Immunology and Infectious Diseases, University of Calgary, Calgary, AB, T2N 4N1, Canada.

[2] BioZone, Department of Chemical Engineering and Applied Chemistry, University of Toronto, Toronto, ON, M5S 1A4, Canada.

[3] University of California Riverside School of Medicine, Biosciences Division

[4] Department of Biochemistry, University of Toronto, Toronto, ON, M5G 1M1, Canada.

[5] Department of Molecular Genetics, University of Toronto, Toronto, ON, M5G 1M1, Canada.

***Corresponding author(s):**

Alexei Savchenko: alexei.savchenko@ucalgary.ca

Adam Godzik: adam.godzik@medsch.ucr.edu

Alexander Ensminger: alex.ensminger@utoronto.ca

Table of Contents

Appendix Figure S1 ………….. 3

Appendix Figure S2 ………….. 4

Appendix Figure S3 ………….. 5

Appendix Figure S4 ………….. 6

Appendix Figure S5 ………….. 7

Appendix Figure S6 ………….. 8

Appendix Figure S7 ………….. 9

Appendix Figure S8 ………….. 10

Appendix Figure S9 ………….. 11

Appendix Figure S10 ………… 12

Appendix Figure S11 ………… 13

Appendix Figure S12 ………… 14

Appendix Table S1 …………… 15

Appendix Table S2 …………… 17

Appendix Table S3 …………… 18

Appendix Table S4 …………… 20

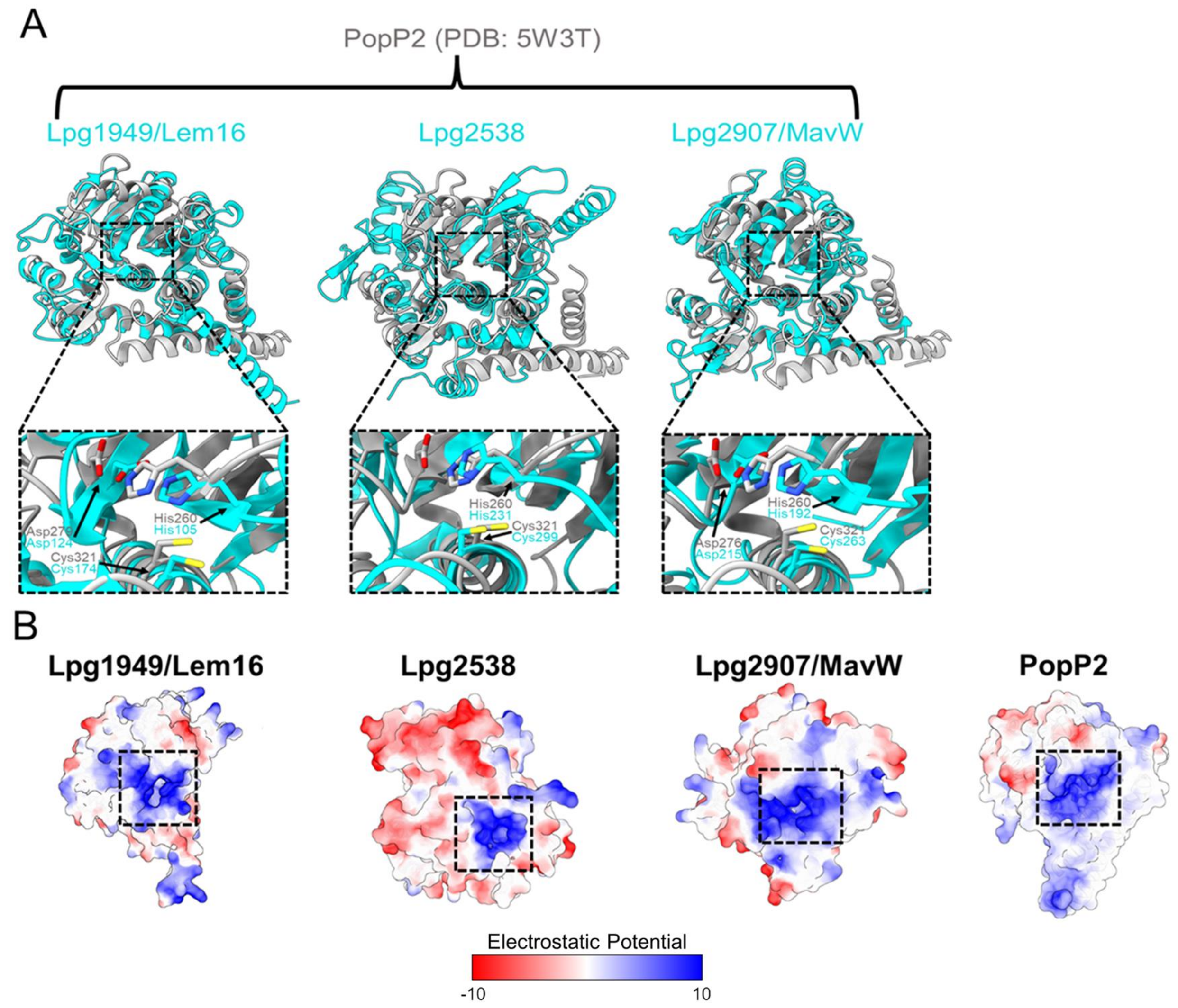

Appendix Figure S1. Identification of *L. pneumophila* effectors that are predicted to be members of the YopJ effector family.

A. Superimpositions of the Lpg1949 (residues 6-327), Lpg2538 (residues 25-463), and Lpg2907 (residues 61-370) models onto the YopJ-like effector, PopP2 (PDB: 5W3T, residues 81-420), from *Ralstonia solanacearum* (Zhang *et al*, 2017) (Top). The zoom-in panels show the putative active site and residues that form the catalytic dyad or triad.

B. Electrostatic potential of the characterized IP6 binding site (boxed) in the PopP2 crystal structure. The corresponding IP6 sites were boxed on the surface representation of the following effectors: Lpg1949/Lem16, Lpg2538, and Lpg2907/MavW. The potential IP6 binding sites were determined by structurally aligning IP6-bound PopP2 with each effector model.

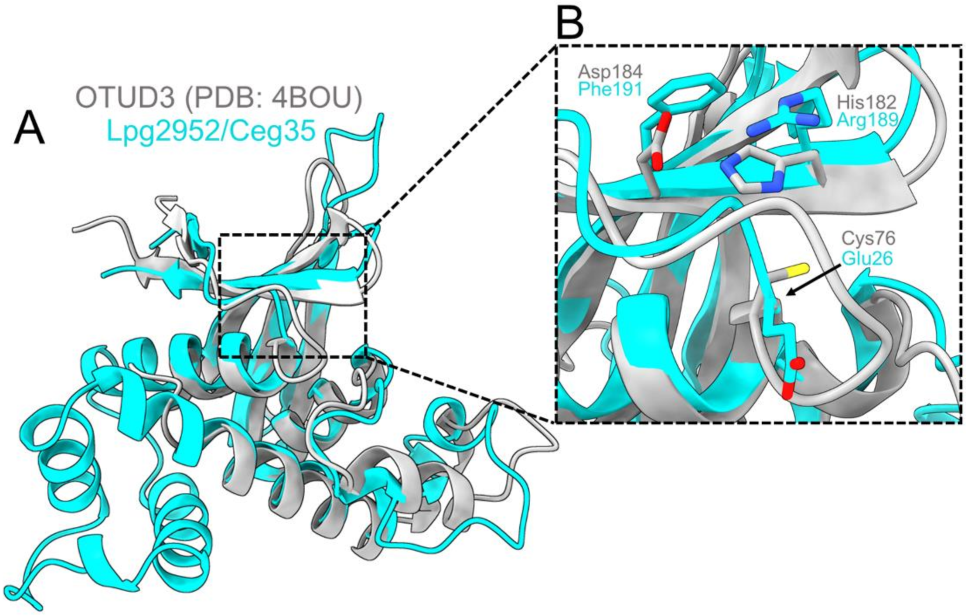

Appendix Figure S2. **Member of the Lot effector family that potentially diverged in function.**

A. Alignment of the Alphafold model of Lpg2952 onto the molecular structure of the human OTU3 enzyme.

B. Zoom in on the catalytic triad of OTU3 (sticks in grey) and the corresponding residues of the Lpg2952 model (sticks in cyan).

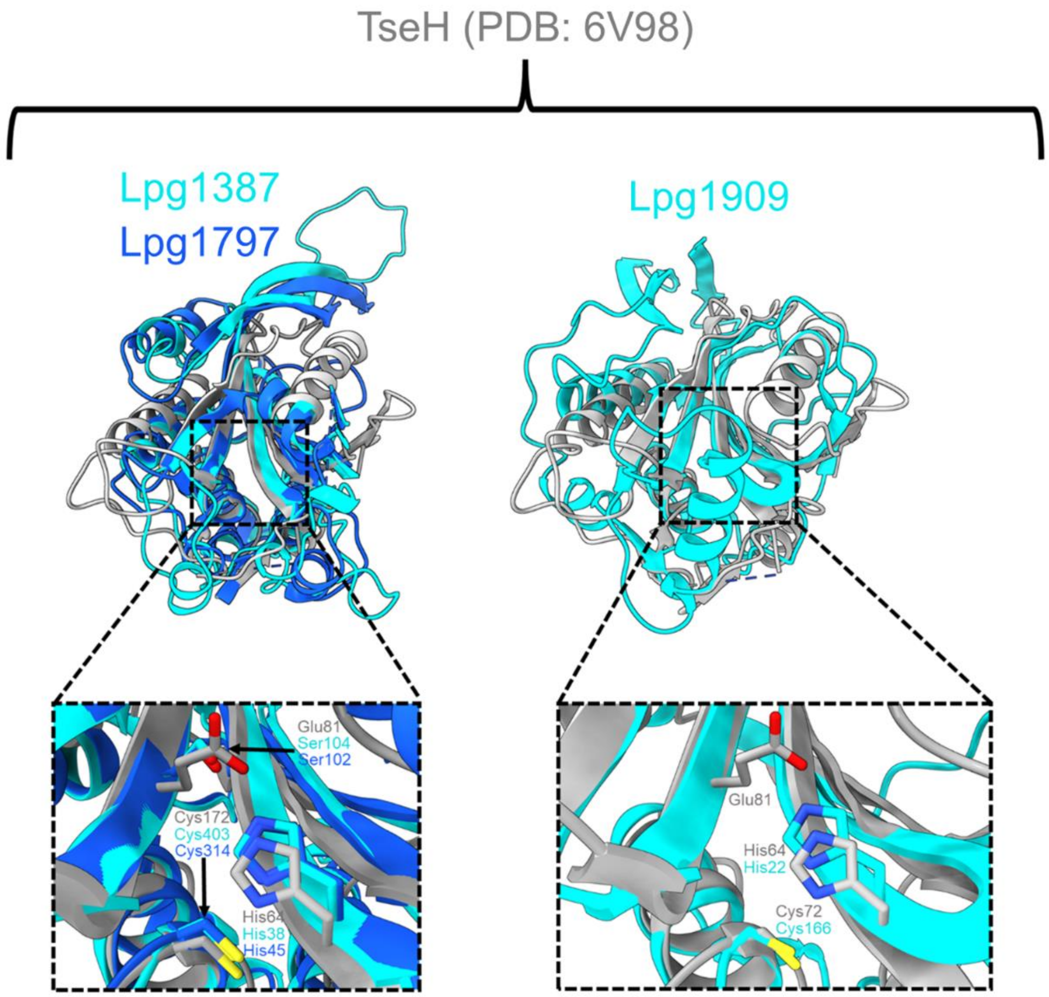

Appendix Figure S3. ***L. pneumophila*** **effectors that display structural similarities to the T6SS effector, TseH, from** ***Vibrio cholerae***

Structural alignments of Lpg1387 (residues 1-139 and 359-462), Lpg1797 (residues 8-126 and 226-384), and Lpg1909 (residues 1-215) models onto the TseH crystal structure (PDB: 6V98, residues 22-223) (Hersch *et al*, 2020). The pop-out is a view of the potential active site harboring residues that could be involved in catalysis (sticks). For both effector models, a serine residue (Ser104 and Ser102, respectively) corresponds to the catalytic glutamate in TseH.

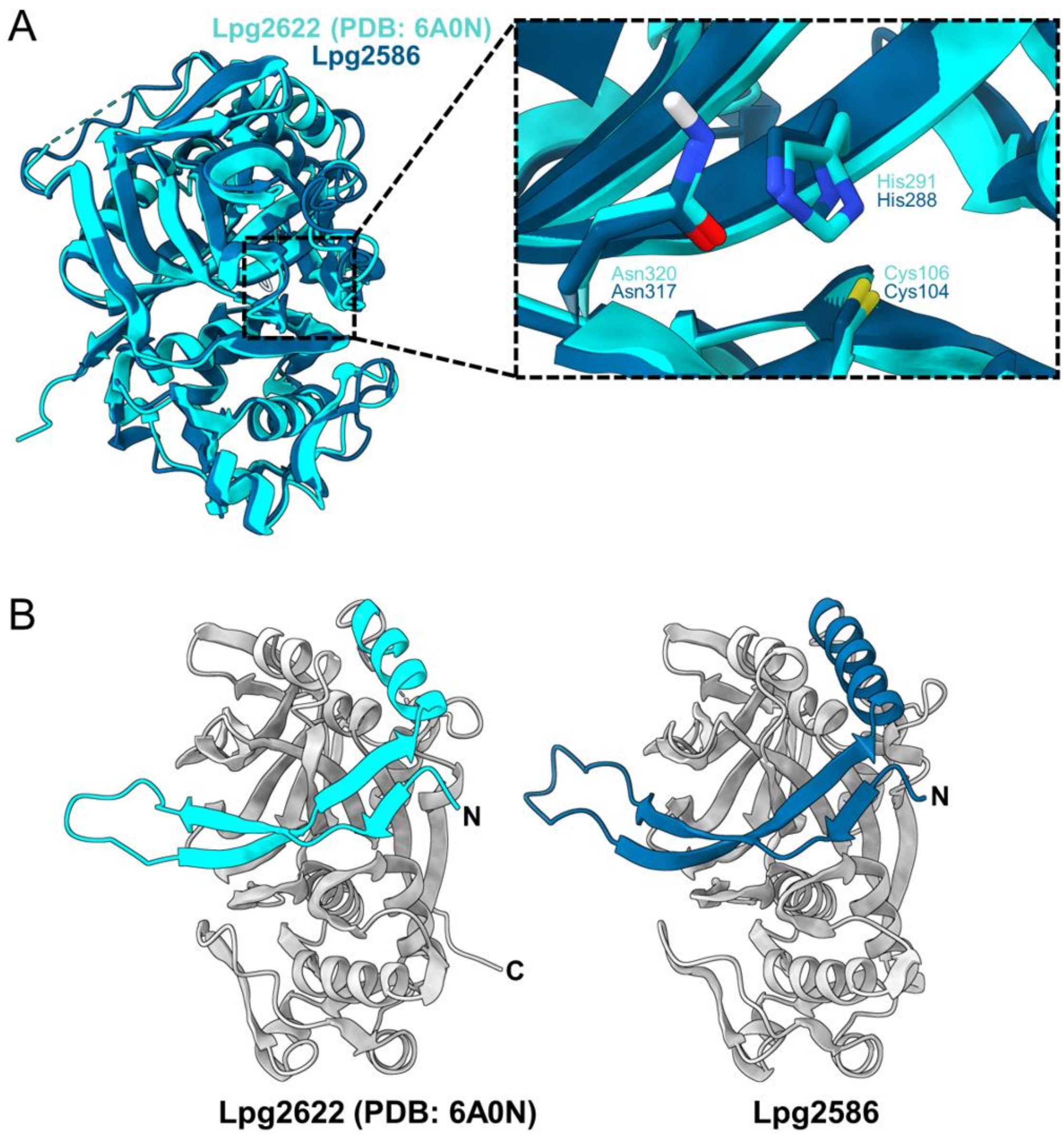

Appendix Figure S4. **Structural similarity between two effectors released by *L. pneumophila* the T2SS and T4SS**

A. Structural alignment of the Lpg2586 model (navy) onto the crystal structure of T2SS *L. pneumophila* effector Lpg2622 (cyan). Inset view of the catalytic residues of Lpg2622 (sticks in cyan) and the corresponding residues of the Lpg2586 model (sticks in navy blue).

B. Colored representation of the novel hairpin-turn-helix motif in the Lpg2622 crystal structure (cyan) and Lpg2586 model (navy).

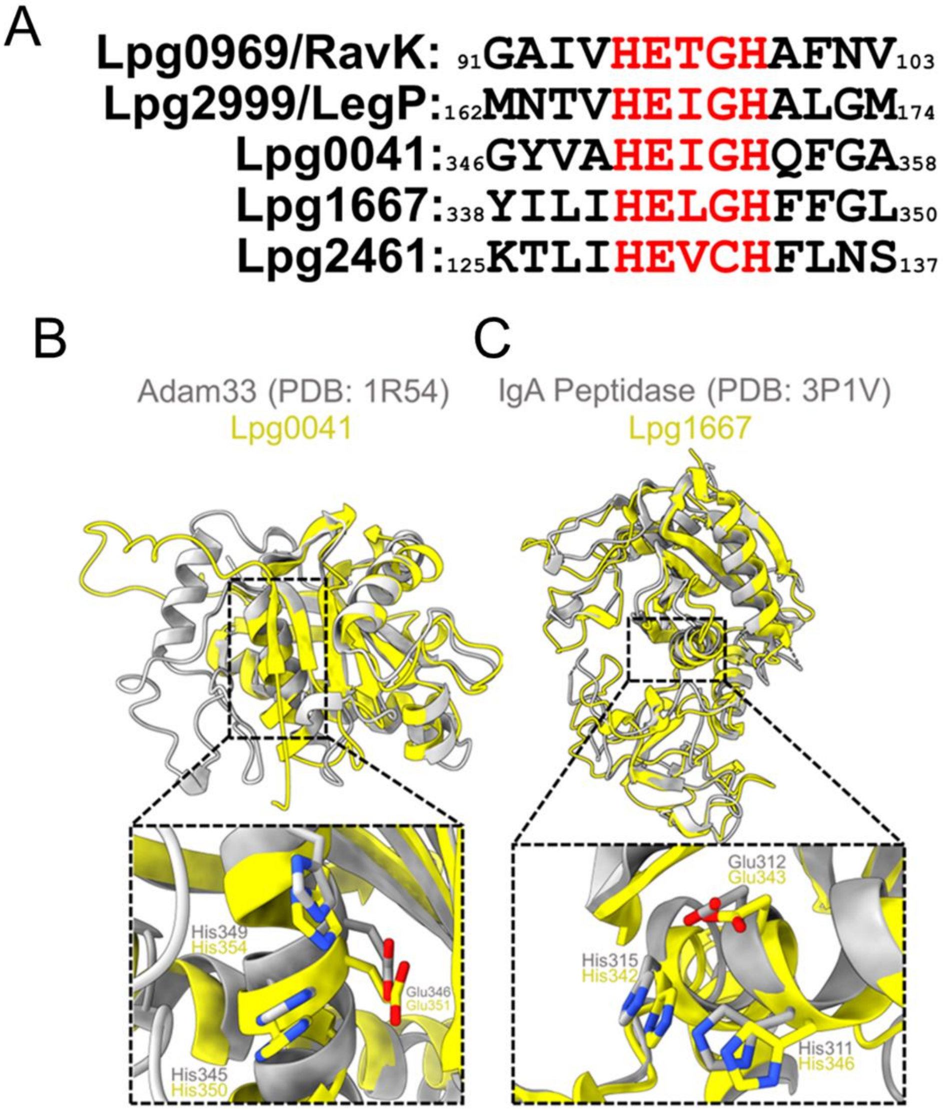

Appendix Figure S5. Expansion of effectors harboring a metalloprotease domain.

A. Sequence alignment of the metalloprotease motif in *L. pneumophila* effectors that were previously identified (Lpg0969/RavK and Lpg2999/LegP) and identified in this study (Lpg0041, Lpg1667, and Lpg2461).

B-C. Superimposition of Lpg0041 (residues 174-351) with human Adam33 (residues 209-409) (Orth *et al*, 2004) and Lpg1667 (residues 177-455) with IgA peptidase from *Bacteroides ovatus* (residues 159-425). Below is a close view of the potential catalytic residues (yellow sticks) of each effector model determined by their top structural hit (grey sticks) from our FATCAT analysis.

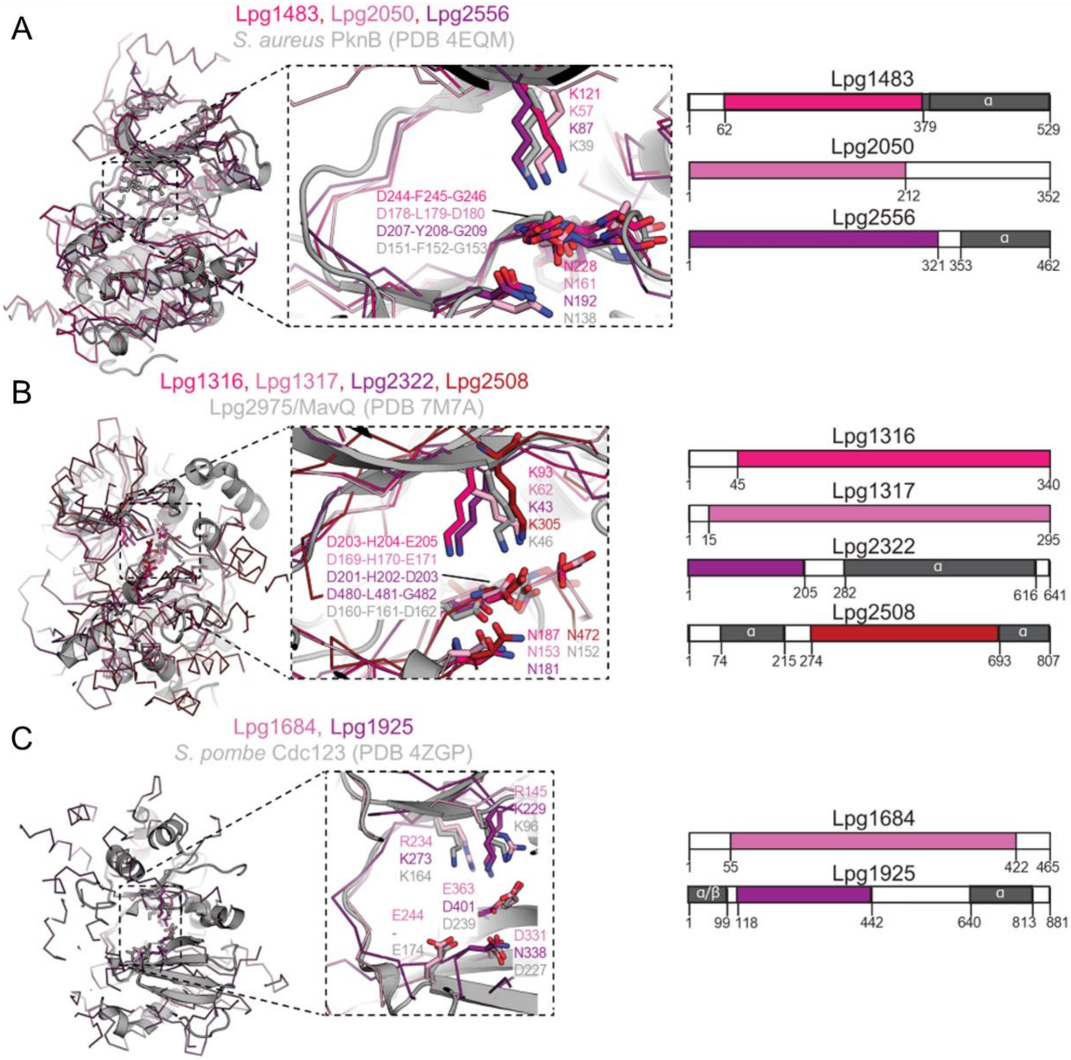

Appendix Figure S6. ***L. pneumophila*** **effector kinases are the second largest functional group of effectors in the arsenal**

A. Putative protein kinases, B. putative small molecule kinases, and C. putative ATP grasp fold kinases. For each panel, left = overlay of effector kinase domains (shades of purple/pink) with a characterized, reference structure from the PDB in grey. Right = Primary domain architecture, with kinase domain colored in shades of purple/pink and alpha-helical regions colored grey.

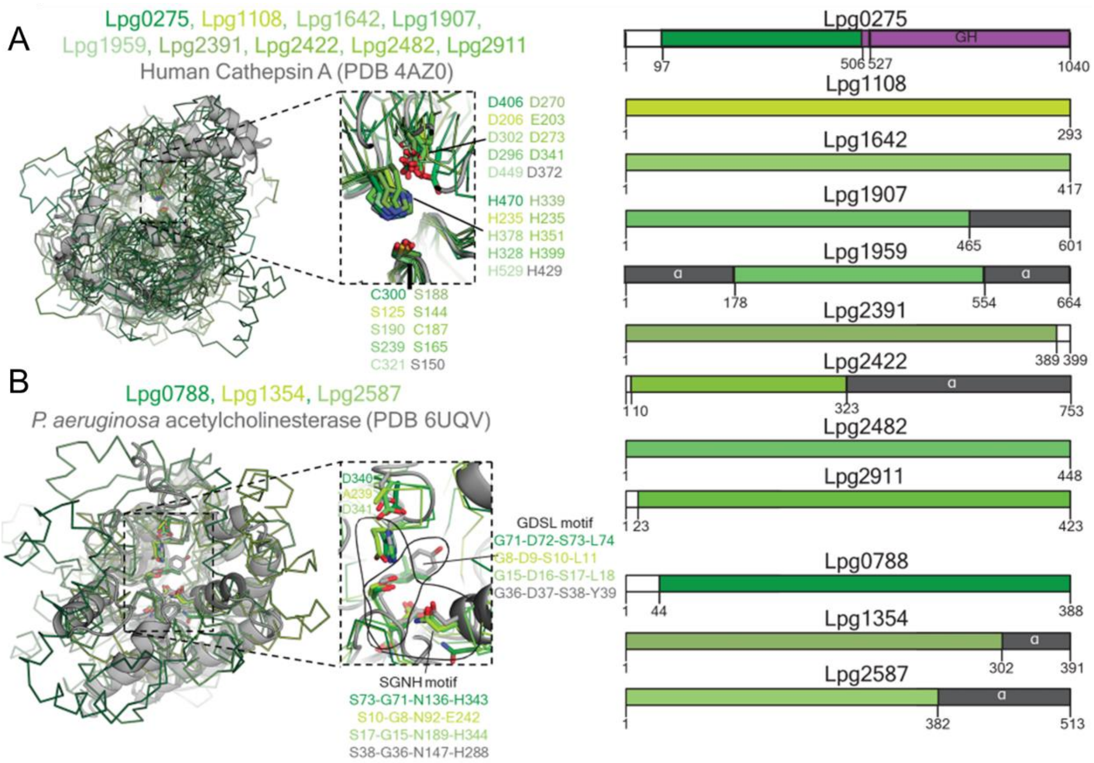

Appendix Figure S7. Structural overlay of *L. pneumophila* effector hydrolases

A. Putative effector hydrolases and B. putative effector hydrolases in the SGNH class. For each panel, left = overlay of effector hydrolase domains (shades of green) with a characterized, reference structure from the PDB in grey. Right = Primary domain architecture, with the hydrolase domain colored in shades of green, alpha-helical regions colored grey, and glycoside hydrolase (GH) domain in Lpg0275/SdbA colored purple.

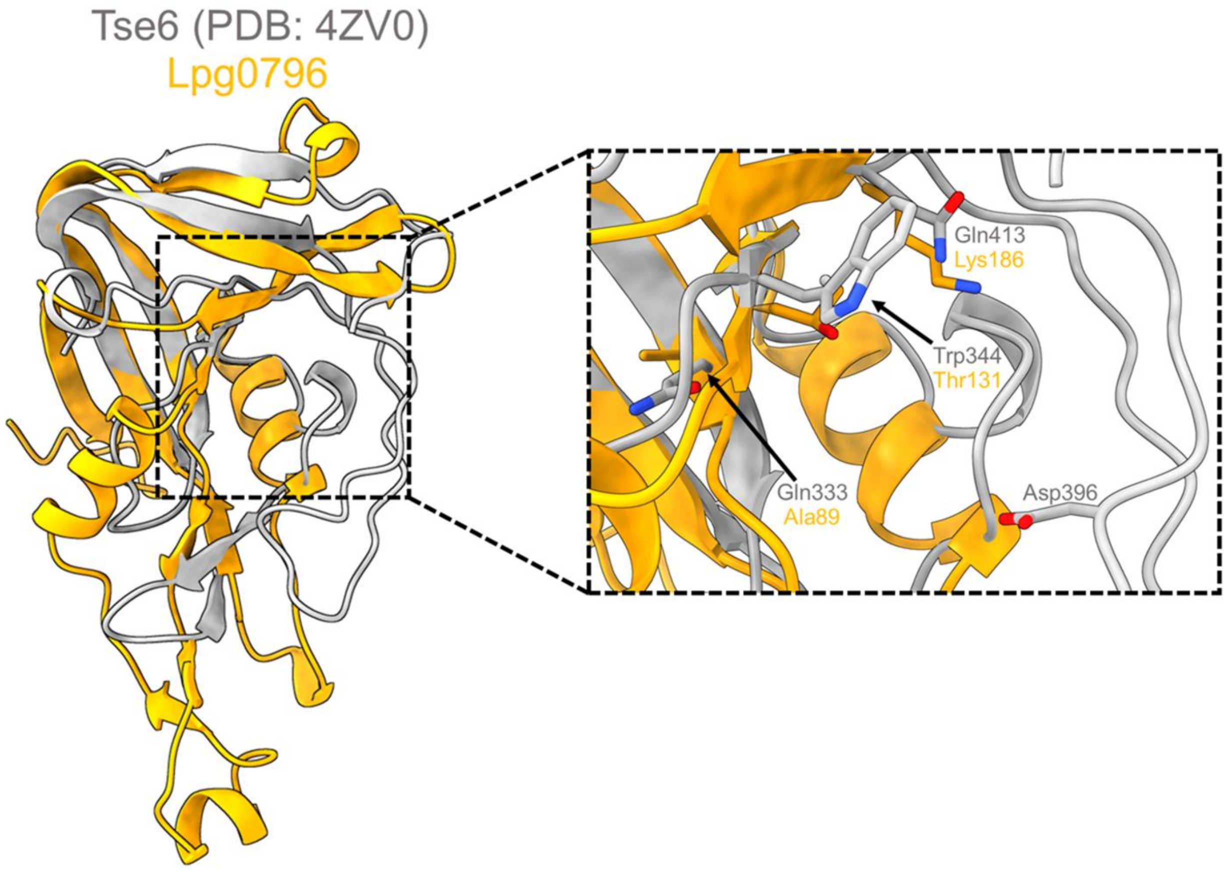

Appendix Figure S8. Uncovering a previously unrecognized ADP-ribosyltransferase fold that lacks residues for catalysis

Structural alignment between Lpg0796 (residues 71-208) and the T6SS effector, Tse6 (residues 318-427), from *P. aeruginosa* (Whitney et al, 2015), followed by an inset view comparing the catalytic residues of Tse6 (grey sticks) with the corresponding residues of Lpg0796 (gold sticks).

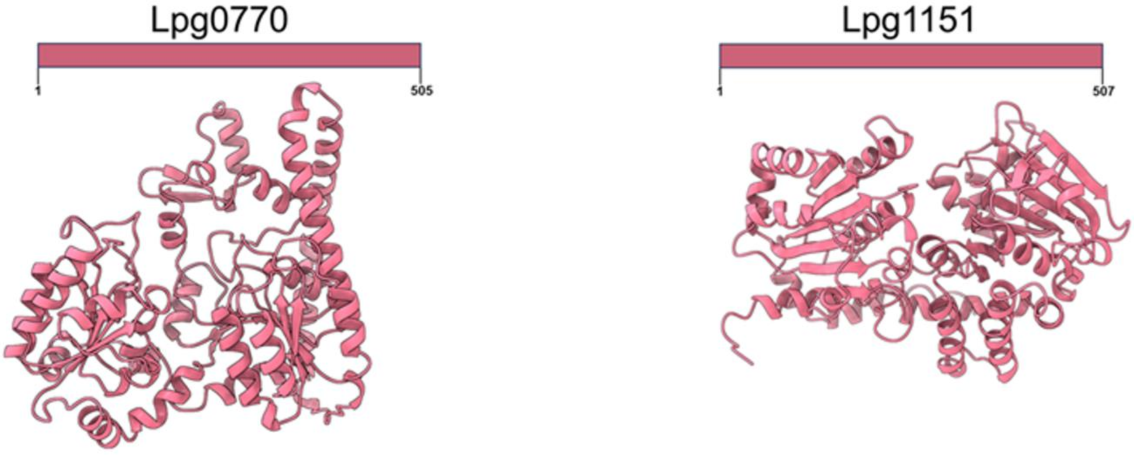

Appendix Figure S9. Identification of *L. pneumophila* effectors glycosyltransferase domains

Structural domain architecture of the GT-B domain (pink) in two *L. pneumophila* effectors.

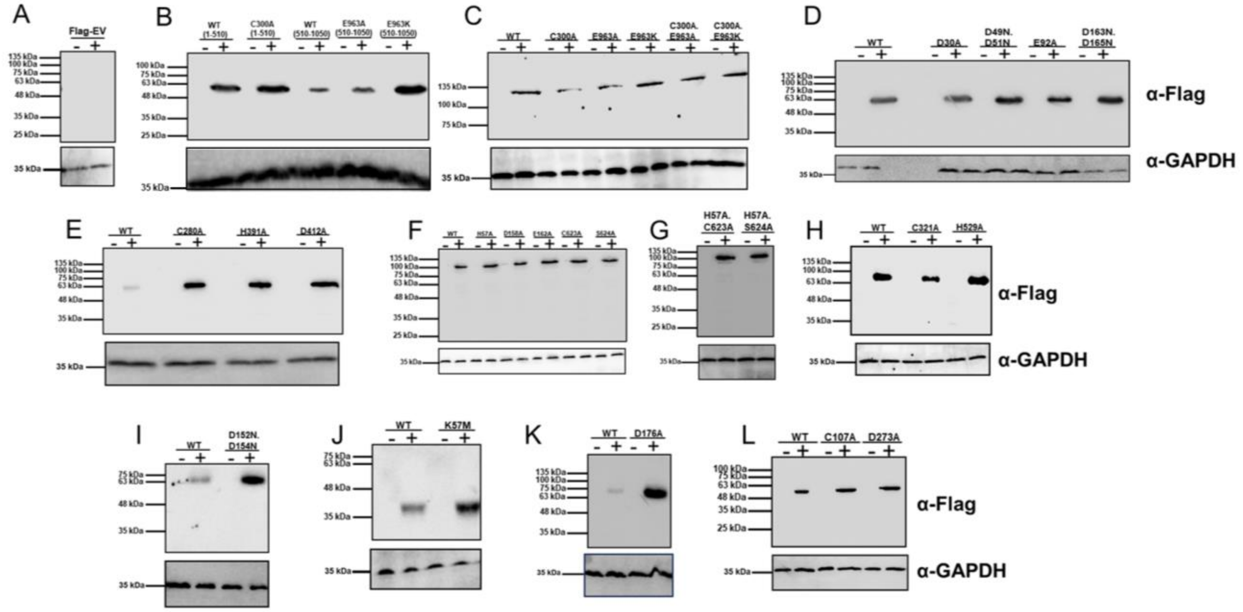

Appendix Figure S10.

Analysis of the expression levels of (A) FLAG-EV, (B) Lpg0275/SdbA fragments, and (C) Lpg0275/SdbA Full length, (D) Lpg0402/LegA9, (E) Lpg1290/Lem8, (F and G) Lpg1355/SidG, (H) Lpg1959, (I) Lpg1961, (J) Lpg2050, (K) Lpg2322/AnkK/LegA5, and Lpg2482/SdbB and their mutants in the *S. cerevisiae* BY4741 strain. “-” indicates yeast incubated with dextrose SD media (non-inducing). “+” indicates yeast incubated with galactose SD media (inducing). FLAG-tagged effector proteins were detected by immunoblotting with a FLAG-tag specific antibody. *S. cerevisiae* GAPDH was detected using a GAPDH-specific antibody for loading control westerns. (A) and (B) were processed on the same blot. (F) and (G) were processed on separate blots.

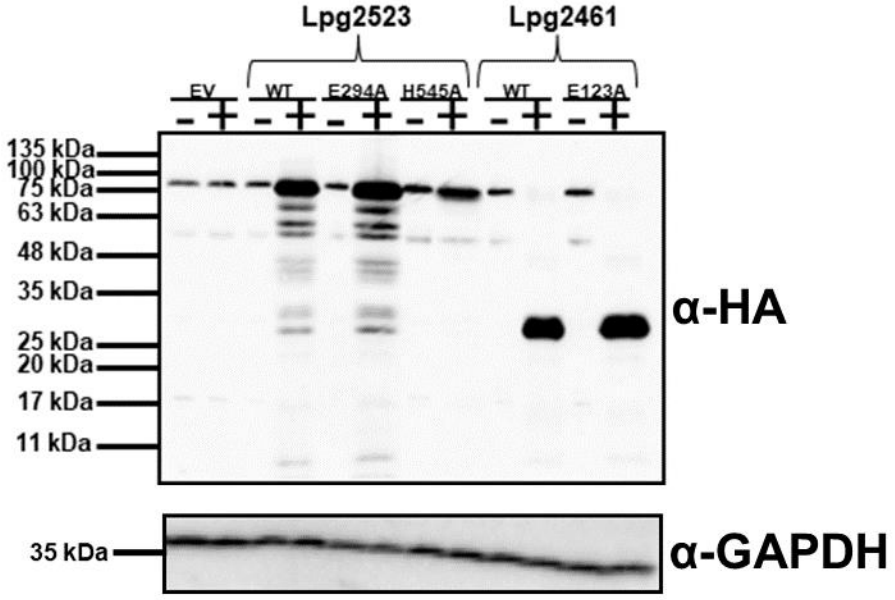

Appendix Figure S11.

Assessment of the expression levels of Lpg2523/Lem26 and Lpg2461 and their respective mutants in the *S. cerevisiae* BY4741 strain. Yeast cells were grown in either non-inducing SD media (dextrose, “-”) or inducing SD media (galactose, “+”). An HA-tag specific antibody was used for the detection of the overexpressed HA-tagged effector protein in each sample. A GADPH-specific antibody was used in loading control westerns.

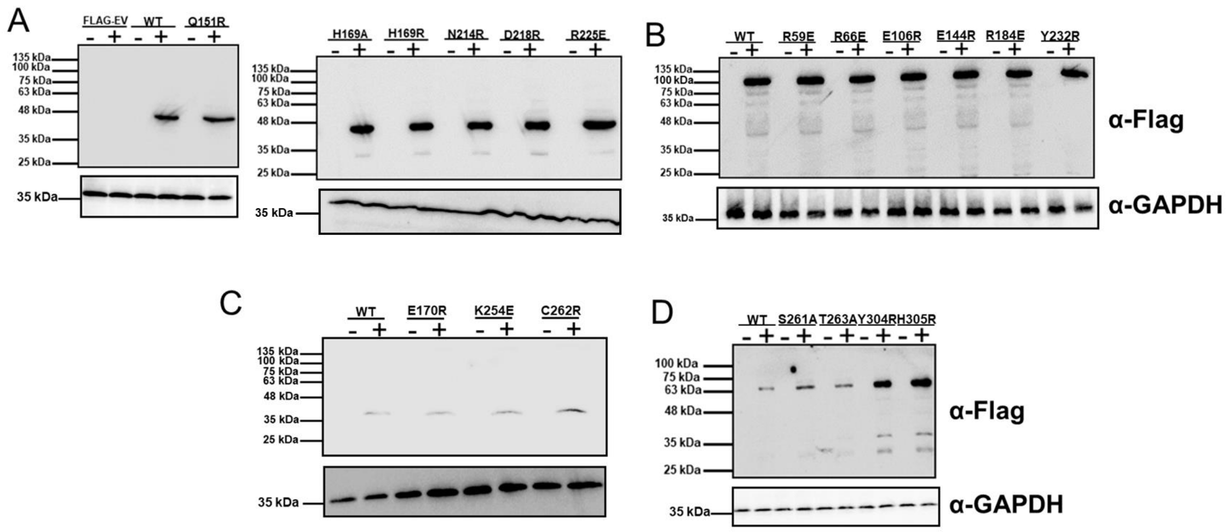

Appendix Figure S12.

Samples of cryptic domain containing effectors and their respective mutants that were transformed in the *S. cerevisiae* BY4741 strain and were grown in dextrose-supplemented SD media (repressing, "-") or galactose-supplemented SD media (inducing, "+") were analyzed by Western blot using a FLAG-tag specific antibody. The loading control westerns were performed using a GADPH-specific antibody. (A) FLAG-EV and Lpg1154/RavQ, (B) Lpg1426/VpdC, (C) Lpg1489/RavX, and (D) Lpg2527/LnaB. FLAG-EV and Lpg1154 mutants were processed on separate western blots.

Appendix Table S1. Homologs of Lpg1154/RavQ that are present in other bacterial species.

Bacteria	Protein Name	Sequence Similarity to Lpg1154/RavQ (%)	Amino acid Boundary of the Unique fold in Homolog	Equivalent Residues of the Proposed Active Site of the Lpg1154/RavQ Unique fold in Homologs
Aquicella siphonis	AQUSIP_02370	55.5	1-369	Gln155-His173-Asn219-Asp223-Glu226-Arg232
Coxiella burnetti	COB66_01075	40.4	89-378	Gln188-His202-Asn247-Asp250-Glu254-Arg265
Candidatus Anoxychlamydiales	K1060chlam5_00018	29.1	1-306	Gln123-His137-Asn184-Asp187-Asp191-Arg200
Estrella lausannensis	ELAC_1467	28.1	19-377	Gln137-His150-Asn194-Asp198-Glu201-Arg205

Bacteria	Protein Name	Sequence Similarity to Lpg1154/RavQ (%)	Amino acid Boundary of the Unique fold in Homolog	Equivalent Residues of the Proposed Active Site of the Lpg1154/RavQ Unique fold in Homologs
Parachlamydia acanthamoebae	DB43_AA00830	26.6	1-420	Gln117-His142-Asn184-Asp187-Asp191-Arg199
Waddlia chondrophila	Wcw_1792	20.4	34-297	Gln105-His121-Asn175-Asp178-Asp182-Arg192

Appendix Table S2. Homologs of Lpg2527/LnaB present in other bacterial species.

**Bacteria**	**Protein Name**	**Sequence Similarity to Lpg2527/LnaB (%)**	**Amino acid Boundary of the Unique fold in Homolog**	**Equivalent Residues of the Proposed Active Site of the Lpg2527 Unique fold in Homologs**
Aquicella siphonis	AQUSIP_08150	21.3	266-623	Ser513-His557-Glu561
Candidatus Berkiella	CC99x_00179	24.2	1-368	Ser254-His298-Glu302
Pseudomonas chlororaphis	C4K37_3919	26.7	1-434	Ser374-His416-Glu420
Pseudomonas antarctica	A7J50_5968	26.8	96-328	Ser386-His430-Glu434
Vibrio caribbeanicus	VIBC2010_01453	21.0	33-487	Ser542-His563-Glu567
Vibrio cholerae strain A325	ERS013201_03035	22.1	136-490	Ser203-His242-Glu246

Vibrio cholerae strain IDH06781	EYB64_18120	23.3	119-490	Ser203-His353-Glu357
Vibrio parahaemolyticus	MAVP-RPIeRC_00038	22.0	119-471	Ser315-His354-Glu358

Appendix Table S3. Primers used for the site-directed mutagenesis of yeast overexpression constructs of cryptic enzymatic domains.

Effector Gene	Mutation	Sequence (5'-->3') of Forward Primer	Sequence (5'-->3') of Reverse Primer	Reference
lpg0275	C300A	TGATGGTATGGCTATAGGTGGGGCG	AGGCAGATGTTCTTAGGG	This study
lpg0275	E963A	AAGTGTCATGGCACAAATGGCCA	AAACCACCTCCACGGATA	This study
lpg0275	E963K	N/A	N/A	(Urbanus *et al*, 2024)
lpg0402	D19A	GCTAAAGGCGCTTTTGCTCTG	AGTTGAATTAACACAATTAGC	This study
lpg0402	D49NxD51N	GATTTAGTTAGAGAAAAAACTTCTGATAATATCAATGTGGTTTTAACCTCTAC	GTAGAGGTTAAAACCACATTGATATTATCAGAAGTTTTTTCTCTAACTAAATC	This study
lpg0402	E92A	GCTTTCATTGCCGCAAACCGATG	CACCACTTCATTATCTACC	This study
lpg0402	D163xD165N	GTGATCAGCCTAATGTTTATGAGCATTTTAACGAAAACGATTCTCTAATACA	TGTATTAGAGAATCGTTTTCGTTAAAATGCTCATAAACATTAGGCTGATCAC	This study

lpg1290	C280A	GAGTGGTATGGCCCACGGCTTAGC	TTTAAACCCATGCCTGGAAG	This study
lpg1290	H391A	GCTATGCTAATAGCAGTTCTGGAGCTGTTGTTGCTATCCACAAAC	GTTTGTGGATAGCAACAACAGCTCCAGAACTGCTATTAGCATAGC	This study
lpg1290	D412A	GTTTCATCATCGACTATTTTGCTGCTAATGCAGGTTGGATGC	GCATCCAACCTGCATTAGCAGCAAAATAGTCGATGATGAAAC	This study
lpg1355	H57A	GGATGTAGGTGCAGCTTCCATTAATATGAAATTAC	CCACCAAAAAATTGCTGATC	This study
lpg1355	D158A	TACAGAAGATGCAATGGTTTGGGAAAG	TTCTGTAATCTTCCCGGC	This study
lpg1355	E162A	CATGGTTTGGGCAAGAGAGGGAA	TCATCTTCTGTATTCTGTAATCTTC	This study
lpg1355	C623A	TACCAATAACGCATCACTTACTTCGATAGAAG	TGTAAATTGAATTCTTTTTTTTCTG	This study
lpg1355	S624A	CAATAACTGCGCACTTACTTC	GTATGTAAATTGAATTCTTTTTTTTC	This study
lpg1959	C321A	GTATGGAATGGCCGGCGGGGCTG	AGTACAATTTTTTTGTTTTTTTGTC	This study
lpg1959	H529A	CCATCACTAATCGCCCTGTAGATTTAGCCAGTAAAAAACTTTACACGC	GCGTGTAAAGTTTTTTACTGGCTAAATCTACAGGGCGATTAGTGATGG	This study
lpg1961	D152Nx D154N	CAATATCGTTTTTTATTTGGATGAAAAC	GTATTGAAGTAATAACCGCCCTC	This study
lpg2050	K57M	TATGTTGTTATGCAACCTAGACCAGATAAAG	TTTAGGAGGCTGGTTTTG	This study

lpg2322	D176A	GAAGAAGACGCTTTG CATAAAG	CAAAGTGTACGAAG TAGC	This study
lpg2461	E130A	TTAATCCATGCAGTCT GTCATTTTTTAAATTC	TGTTTTGGCAATTTC TTCAAC	This study
lpg2482	C187A	CATCATTTTACAGGGT GATGCCTACGGAGCA AGCATTGCA	TGCAATGCTTGCTC CGTAGGCATCACCC TGTAAAATGATG	This study
lpg2482	D273A	CACTTGGGAGCTCAA ACGCTAG	TTGAATATGACATTG GTAAGG	This study
lpg2523	E294A	CCAGGAGCGATTAAA AATGCACGCTCAGCA GGATAAC	GTTATCCTGCTGAG CGTGCATTTTTAATC GCTCCTGG	This study
lpg2523	H545A	TAGACCAAATGCTGG GCTGTCTC	TGAAGAGTTTTATTG TTACC	This study

Appendix Table S4. Primers used in this study for the site-directed mutagenesis of plasmids that overexpress effectors containing unique folds/domains in yeast.

**Effector Gene**	**Mutation**	**Sequence (5'-->3') of Forward Primer**	**Sequence (5'-->3') of Reverse Primer**	**Reference**
lpg1154	Q151R	CAAATGGTTATACCG ATCTTCGGCATGGCA GGGGGCGTAATAC	GTATTACGCCCCCTG CCATGCCGAAGATC GGTATAACCATTTG	This study
lpg1154	H169A	CATAACTATCACTGA AGCCTGTGCTAGCAG TCTTACTCCATCATTG	CAATGATGGAGTAAG ACTGCTAGCACAGG CTTCAGTGATAGTTA TG	This study
lpg1154	H169R	CTATCACTGAAGCCT GTCGTAGCAGTCTTA CTCCATC	GATGGAGTAAGACT GCTACGACAGGCTT CAGTGATAG	This study

lpg1154	N214R	GGTAGAATTACCTCCCTTTGTGAGGGATTTCGATGATATCCTGGAAAAG	CTTTTCCAGGATATCATCGAAATCCCTCACAAAGGGAGGTAATTCTACC	This study
lpg1154	D218R	CCCTTTGTGAATGATTTCGATCGTATCCTGGAAAAGGCTTGCAG	CTGCAAGCCTTTTCCAGGATACGATCGAAATCATTCACAAAGGG	This study
lpg1154	E221R	GTGAATGATTTCGATGATATCCTGAGAAAGGCTTGCAGGAAAAAATGC	GCATTTTTTCCTGCAAGCCTTTCTCAGGATATCATCGAAATCATTCAC	This study
lpg1154	R225E	GATATCCTGGAAAAGGCTTGCGAGAAAAAATGCTTGGAAATCCTG	CAGGATTTCCAAGCATTTTTTCTCGCAAGCCTTTTCCAGGATATC	This study
lpg1426	R59E	GGAAATTATTTATTTGATGATGAAGAGATGATTTTTGATTTCACTCGATTGAG	CGAGTGAAATCAAAAATCATCTCTTCATCATCAAATAAATAATTTCCAAG	This study
lpg1426	R66E	GTATGATTTTTGATTTCACTGAGTTGAGTGATTCAAAAAGAGCTTTATTTATTG	GCTCTTTTTGAATCACTCAACTCAGTGAAATCAAAAATCATACGTTCATC	This study
lpg1426	E106R	GAATATCGAGGTTTCACTGCGAGGGTTGCTTTAAGTTGGTGG	CCACCAACTTAAAGCAACCCTCGCAGTGAAACCTCGATATTC	This study
lpg1426	E144R	CAATTATCAGCTGACTGGTATTAGAATGTGTCATGGAAAGCAGGG	CCCTGCTTTCCATGACACATTCTAATACCAGTCAGCTGATAATTG	This study
lpg1426	R184E	TAGCCAAAGTGAGCCATTAGGTAATGTTAAAGAGGTATTCATTACTGATAAACTGGTTG	CAACCAGTTTATCAGTAATGAATACCTCTTTAACATTACCTAATGGCTCACTTTGGCTA	This study
lpg1426	Y232R	GCTCGTCATAAAGACATGTATGACCGCAGA	CCATTGAAACGTTGCATTTTTCTGCGGTCA	This study

		AAAATGCAACGTTTCAATGG	TACATGTCTTTATGACGAGC	
lpg1489	E170R	CGTAATGGAGTGATGCTTGCTATAAGATTGCACGAAGATGCCG	CGGCATCTTCGTGCAATCTTATAGCAAGCATCACTCCATTACG	This study
lpg1489	K254E	CTATTTGAGCTAATACGTTCTGTAGAGGTCCTGGGTAACAATG	CATTGTTACCCAGGACCTCTACAGAACGTATTAGCTCAAATAG	This study
lpg1489	C262R	GTCCTGGGTAACAATGGCAGTCGTAAAACTATTTATCATTGCTC	GAGCAATGATAAATAGTTTTACGACTGCCATTGTTACCCAGGAC	This study
lpg2527	Y304R	TTCCAGTTATATCAAGGAAGGTCGTCACAGCTACAGTGAGGTTG	CAACCTCACTGTAGCTGTGACGACCTTCCTTGATATAACTGGAA	This study
lpg2527	H305R	TATATCAAGGAAGGTTATCGCAGCTACAGTGAGGTTGTG	CACAACCTCACTGTAGCTGCGATAACCTTCCTTGATATA	This study
lpg2527	S261A	TTGTGGCGTCGCTGGCTGGCACAACCTAC	GTAGGTTGTGCCAGCCAGCGACGCCACAA	This study
lpg2527	T263A	GCGTCGCTGTCTGGCGCAACCTACTCACTTA	TAAGTGAGTAGGTTGCGCCAGACAGCGACGC	This study

References:

Hersch SJ, Watanabe N, Stietz MS, Manera K, Kamal F, Burkinshaw B, Lam L, Pun A, Li M, Savchenko A *et al* (2020) Envelope stress responses defend against type six secretion system attacks independently of immunity proteins. *Nat Microbiol* 5: 706-714

Orth P, Reichert P, Wang W, Prosise WW, Yarosh-Tomaine T, Hammond G, Ingram RN, Xiao L, Mirza UA, Zou J *et al* (2004) Crystal structure of the catalytic domain of human ADAM33. *J Mol Biol* 335: 129-137

Urbanus ML, Zheng TM, Khusnutdinova AN, Banh D, O'Connor Mount H, Gupta A, Stogios PJ, Savchenko A, Isberg RR, Yakunin AF *et al* (2024) A random mutagenesis screen enriched for missense mutations in bacterial effector proteins. *G3 (Bethesda)* 14

Whitney JC, Quentin D, Sawai S, LeRoux M, Harding BN, Ledvina HE, Tran BQ, Robinson H, Goo YA, Goodlett DR *et al* (2015) An interbacterial NAD(P)(+)

glycohydrolase toxin requires elongation factor Tu for delivery to target cells. *Cell* 163: 607-619
Zhang ZM, Ma KW, Gao L, Hu Z, Schwizer S, Ma W, Song J (2017) Mechanism of host substrate acetylation by a YopJ family effector. *Nat Plants* 3: 17115
