# Supplementary material for: Global atlas of predicted functional domains in Legionella pneumophila Dot/Icm translocated effectors
Source: Mol Syst Biol. 2024 Nov 19;21(1):3. doi: 10.1038/s44320-024-00076-z (PMC11696984; doi:10.1038/s44320-024-00076-z)
Supplement: Supplementary file 5 — Expanded View Figures [file 44320_2024_76_MOESM5_ESM.pdf]

Expanded View Figures

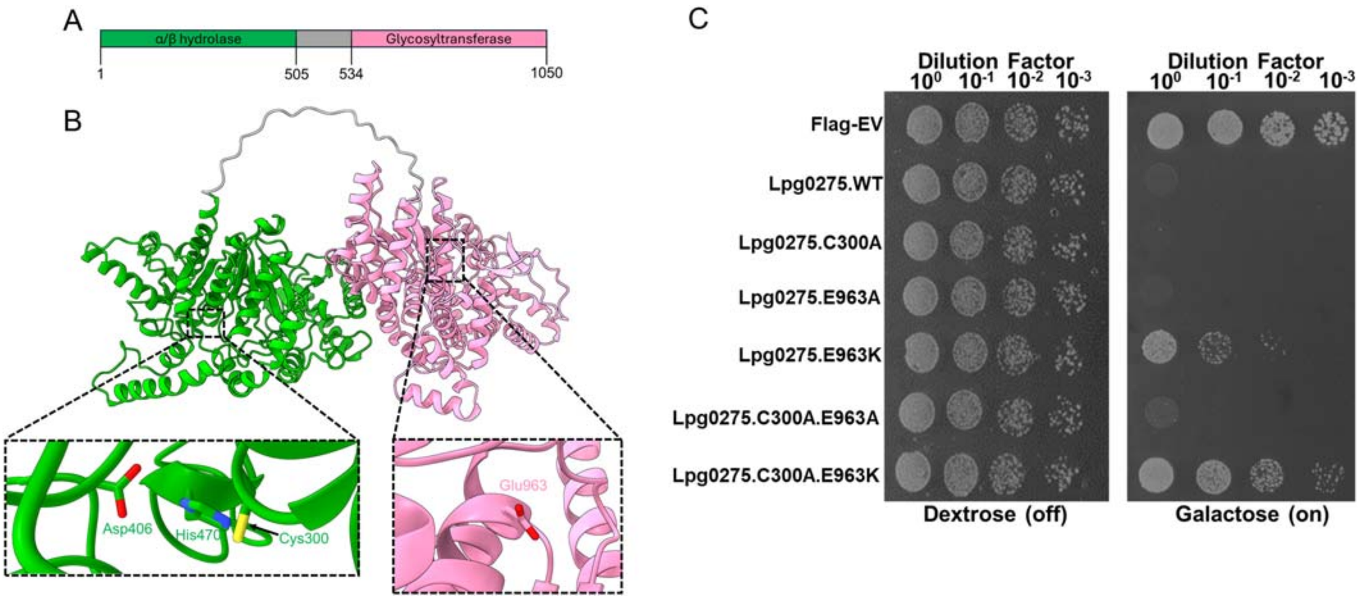

Figure EV1. Both domains of Lpg0275/SdbA are involved in yeast toxicity.

(**A**) Schematic of the domain organization of Lpg0275/SdbA. The N-terminal domain shown in green corresponds to the hydrolase domain, whereas the pink indicates the glycosyltransferase domain. (**B**) Alphafold2 model of Lpg0275/SdbA. The green represents the hydrolase domain, and the pink represents the glycosyltransferase domain. Below is a zoomed-in view of the predicted catalytic residues of each predicted enzymatic domain. (**C**) Yeast toxicity panel of strains expressing FLAG-tagged constructs of full-length wildtype Lpg0275 and its variants.

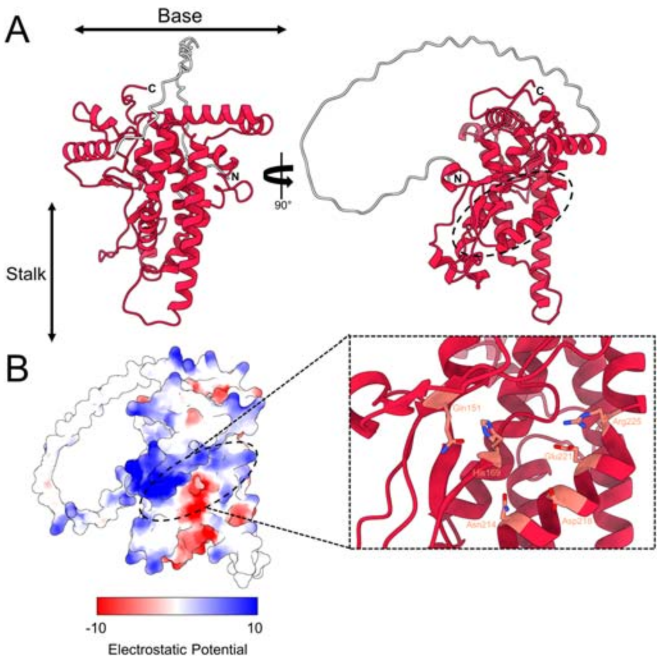

Figure EV2. Lpg1154/RavQ forms a unique "T" shape containing a highly conserved groove that may serve as an active site.

(**A**) The Lpg1154/RavQ model (residues 59–389, red) shows the base and stalk that form the "T" shape. (**B**) An electrostatic potential surface representation of a potential active site cavity of Lpg1154/RavQ, followed by a zoom-in of the conserved residues identified in Lpg1154/RavQ and its orthologs from the *Legionella* genus, which are arranged in a potential active site.

 © The Author(s)

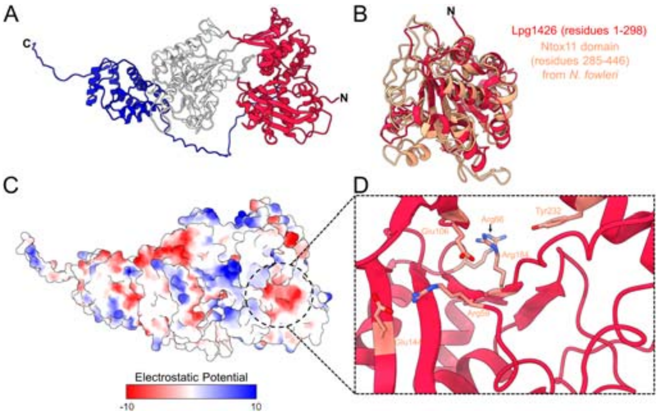

Figure EV3. Lpg1426/VpdC has a cryptic domain on the N-terminus that has structural similarity to the Ntox11 putative toxin found in human pathogenic amoeba.

(**A**) Cartoon representation of the Lpg1426/VpdC Alphafold2 model. The cryptic domain is found on the N-terminus (red), followed by a central phospholipase domain (white) and the C-terminal helical bundle involved in interactions with ubiquitin. (**B**) Structural alignment of the Lpg1426/VpdC cryptic domain (residues 1–298, red) onto the Ntox11 Alphafold2 model (residues 285–446, salmon) from *N. fowleri*. (**C**) Surface representation of the electrostatic potential of the Lpg1426/VpdC model that also shows a conserved negatively-charged pocket (dotted circle) is present in the cryptic domain. (**D**) Zoom in on the positively charged region where highly conserved residues (salmon sticks), which are present in the *Legionella* orthologs of Lpg1426/VpdC, form a pocket.

© The Author(s)

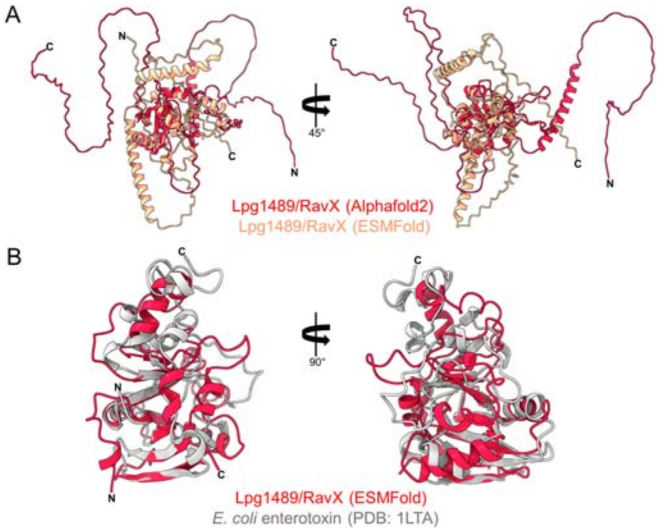

Figure EV4. Lpg1489/RavX has a central globular cryptic domain surrounded by disordered loops.

(**A**) Structural alignment of the full-length Lpg1489/RavX models generated by Alphafold2 (red) and ESMFold (salmon). (**B**) ESMFold model of the Lpg1489/RavX cryptic domain (residues 83–263, red) onto the *E. coli* enterotoxin (PDB: 1LTA, residues 1–181, gray).

 © The Author(s)

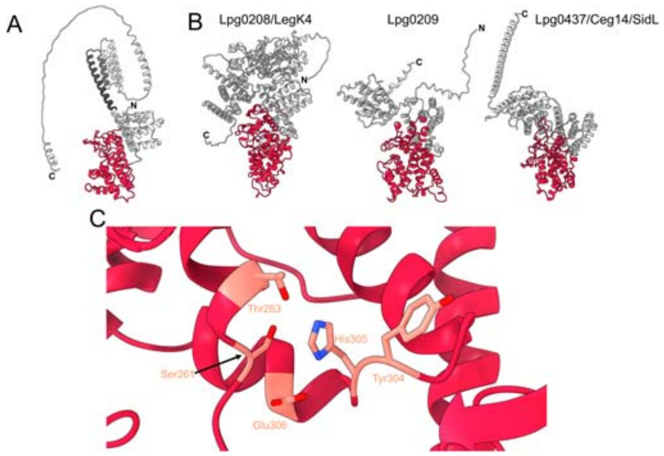

Figure EV5. The cryptic domain of Lpg2527/LnaB is present in other *L. pneumophila* effectors, harboring a conserved set of residues that resemble a potential active site.

(**A**) Alphafold2 model of Lpg2527/LnaB which highlights the cryptic domain (red) and the helical bundle that was previously shown to be important in the activation of the NF-κB pathway (dark gray) (Losick et al, 2010). (**B**) Representation of the Lpg2527/LnaB cryptic domain (shown in red) that is present in other *L. pneumophila* effectors (Lpg0208/LegK4, Lpg0209, and Lpg0437/Ceg14/SidL). (**C**) Zoomed-in image of the putative active site of Lpg2527/LnaB that is also conserved in its *Legionella* orthologs and other *L. pneumophila* effectors containing this domain.

© The Author(s)
